# Laparoscopic hepatectomy for liver metastasis of lung large-cell neuroendocrine carcinoma: A case report

**DOI:** 10.1016/j.ijscr.2019.10.026

**Published:** 2019-10-21

**Authors:** Hisoka Yamane, Sachiko Yoshida, Toshihiko Yoshida, Masayasu Nishi, Takashi Yamagishi, Hironobu Goto, Dai Otsubo, Akinobu Furutani, Taku Matsumoto, Yasuhiro Fujino, Kazuyoshi Kajimoto, Toshiko Sakuma, Masahiro Tominaga

**Affiliations:** a Division of Gastroenterological Surgery, Hyogo Cancer Center, 13-70, Kitaoji-cho, Akashi, Hyogo 673-8558, Japan; b Division of Diagnostic Pathology, Hyogo Cancer Center, 13-70, Kitaoji-cho, Akashi, Hyogo 673-8558, Japan

**Keywords:** LCNEC, large-cell neuroendocrine carcinoma, WHO, World Health Organization, CT, computed tomography, NET, neuroendocrine tumor, SCLC, small cell lung carcinoma, ProGRP, progastrin-releasing peptide, S7, segment 7, FDG-PET, ^18^F-fluorodeoxyglucose positron emission tomography, SUVmax, maximum standardized uptake value, MRI, magnetic resonance imaging, ICG, indocyanine green, Large cell neuroendocrine carcinoma, Liver metastasis, Laparoscopic hepatectomy, Case report

## Abstract

**Introduction:**

Lung large-cell neuroendocrine carcinoma (LCNEC) is an aggressive and a rare type of lung cancer, and the prognosis of LCNEC with distant metastasis is extremely poor, with a five-year survival rate of 0%. Here, we report a case of laparoscopic hepatectomy for liver metastasis of lung LCNEC.

**Presentation of case:**

A 63-year-old man received a routine physical examination, and abnormal chest radiographic findings were observed; chest computed tomography (CT) in our hospital revealed that the patient had left pneumothorax and a lesion measuring 18 mm in the inferior lingular segment of the lung. The patient underwent thoracoscopic lobectomy, and the final pathological diagnosis was lung LCNEC. Four years after surgery, abdominal CT revealed a mass measuring 27 mm in the liver. The patient underwent laparoscopic partial hepatectomy, and postoperative pathological examination showed liver metastasis of LCNEC. There was no sign of recurrence 6 months after hepatectomy.

**Discussion:**

LCNEC with distant metastasis has a poor response to systemic chemotherapy, and the median survival time of patients with distant metastasis is estimated to be approximately 6 months, with a five-year survival rate of 0%. Although the common site of metastasis from LCNEC is the liver, there are no previous reports of hepatectomy for liver metastasis of LCNEC.

**Conclusion:**

We report a case of laparoscopic hepatectomy for liver metastasis of lung LCNEC. It is suggested that surgical resection for solitary distant metastasis of LCNEC may improve prognosis.

## Highlights

• Lung large-cell neuroendocrine carcinoma (LCNEC) is an aggressive and a rare type of lung cancer.
• The prognosis of LCNEC with distant metastasis is extremely poor.
• Surgical resection for liver metastasis of LCNEC may improve prognosis.

## 1 Introduction

In the 2015 World Health Organization (WHO) classification, lung neuroendocrine tumors (NETs) were classified into four categories: small-cell lung carcinoma (SCLC), large-cell neuroendocrine carcinoma (LCNEC), carcinoid tumor and diffuse idiopathic pulmonary neuroendocrine cell hyperplasia. LCNEC is an aggressive and a rare type of lung cancer that accounts for 3 % of all primary lung malignancies [1]. Currently, LCNEC is classified as a variant of large cell carcinoma; however, the clinical and biological characteristics are similar to those of SCLC. Therefore, there is still no consensus on the treatment strategy for LCNEC [2]. Patients with LCNEC have poor prognoses with five-year survival rates of 15–57% and distant metastasis rates of 65% [3]. LCNEC with distant metastasis has a poor response to systemic chemotherapy [4], and the life expectancy of the patient is estimated at approximately 6 months [5]. The common site of metastasis from LCNEC is the liver [6], but there are no previous reports of hepatectomy for liver metastasis of LCNEC. Herein, we present a case report to perform laparoscopic hepatectomy for liver metastasis from lung LCNEC. This report has been reported in line with the SCARE guideline [7].

## 2 Presentation of case

**Fig. 1:**
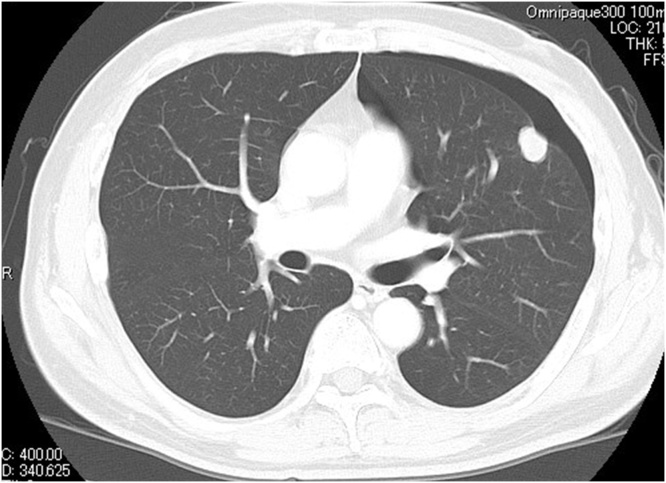
Chest CT showing left pneumothorax and a lesion measuring 18 mm in diameter in the inferior lingular segment of the lung.

**Fig. 2:**
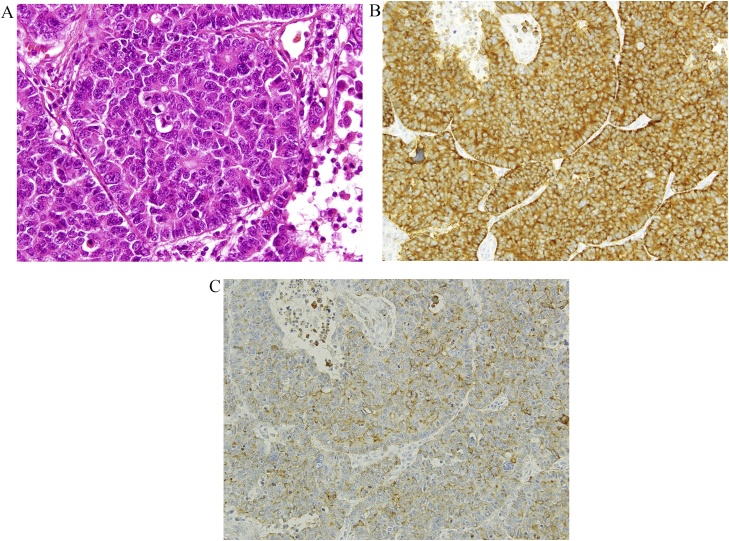
Pathological findings of the lung. A) hematoxylin-eosin staining. Magnification 400×. B) chromogranin A staining. Magnification 200×. C) synaptophysin staining. Magnification 200×.

**Fig. 3:**
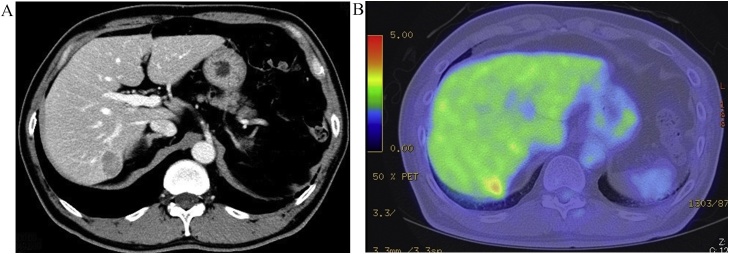
A) Abdominal CT showing a mass measuring 27 mm in diameter in S7 of the liver. B) FDG-PET showing high accumulation of FDG with an SUVmax 3.4 in the liver tumor.

**Fig. 4:**
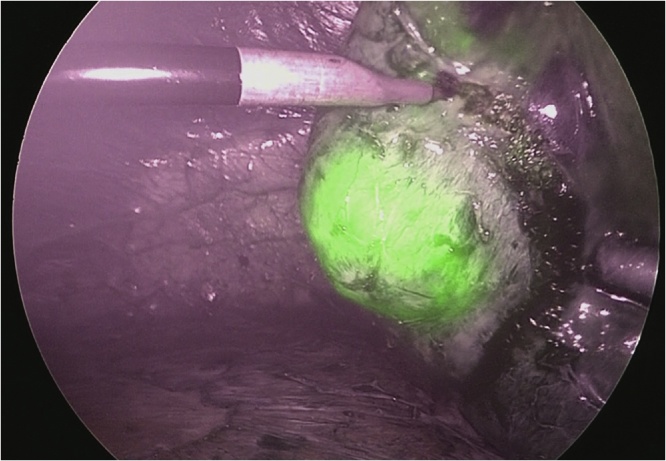
Intraoperative fusion ICG-fluorescence imaging.

**Fig. 5:**
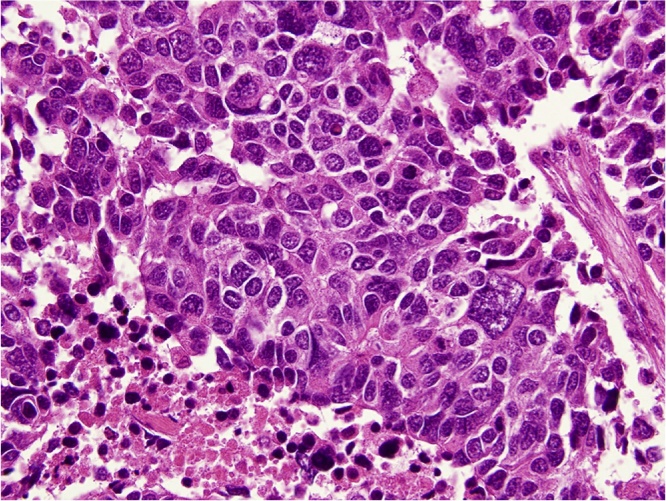
Pathological findings of the liver (hematoxylin-eosin staining. Magnification 400×).

A 63-year-old man was diagnosed with abnormal chest radiographic findings by routine physical examination and was referred to the Department of Thoracic Surgery at our hospital. He had no past medical and surgical history. Chest CT revealed left pneumothorax and a lesion measuring 18 mm in diameter in the inferior lingular segment of the lung (Fig. 1). The patient underwent thoracoscopic lobectomy with the presumed diagnosis of lung cancer. He had a favorable clinical course without any complications, and he was discharged on postoperative day 9. Histopathological examination of the resected specimen stained with hematoxylin and eosin revealed neuroendocrine morphology, such as organoid nesting, rosette-like structures and peripheral palisading. The tumor cells generally had a polygonal shape and moderate cytoplasm. Nucleoli were sometimes prominent (Fig. 2a). Mitotic counts were 90 cells in mitosis per 2 mm^2^ of tissue. Moreover, staining of the tumor was positive for chromogranin A (Fig. 2b) and synaptophysin (Fig. 2c), which are neuroendocrine markers. The final pathological diagnosis was lung LCNEC, and T1bN0M0, stage IA2, based on the Union for International Cancer Control classification (eighth edition). Subsequently, the patient received four courses of adjuvant chemotherapy with cisplatin and VP-16. Four years after surgery, the level of progastrin-releasing peptide (ProGRP), a specific neuroendocrine tumor marker, was elevated and dynamic contrast-enhanced CT revealed a mass measuring 27 mm in diameter in segment 7 (S7) of the liver (Fig. 3a). ^18^F-fluorodeoxyglucose positron emission tomography (FDG-PET) revealed a high accumulation of FDG with a maximum standardized uptake value (SUVmax) of 3.4 in the liver tumor (Fig. 3b), and no other high uptake value. Brain magnetic resonance imaging (MRI) showed no metastasis. To avoid the risk of peritoneal dissemination from the liver tumor, we did not perform preoperative liver biopsy. Our preoperative diagnosis was solitary liver metastasis from lung LCNEC, and we planned to perform indocyanine green (ICG) fluorescence-navigated laparoscopic S7 partial hepatectomy. ICG was intravenously injected at a dose of 0.5 mg/kg as a routine measure for the evaluation of liver function, 24 h preoperatively. The procedures were performed using a pressure-controlled carbon dioxide pneumoperitoneum, which was maintained below 10 mmHg. After the right lobe was mobilized, fusion ICG-fluorescence imaging indicated that a green pseudocolor tumor was present on the liver surface of S7 (Fig. 4). Using the crush-clamp method with a harmonic scalpel (Ethicon, USA), the liver parenchyma was transected. Pringle’s maneuver was performed by clamping the hepatoduodenal ligament using the tourniquet method for 15 min followed by a 5-min release period, and a total of six temporary clamps were performed during parenchymal resection. The operative time was 331 min and the estimated intraoperative blood loss was 10 ml. On gross appearance, the surgical margin of the tumor was within the cut surface. The resected tumor was pathologically similar to the primary lung LCNEC (Fig. 5) and staining of the tumor was positive for chromogranin A and synaptophysin. Postoperative pathological examination showed liver metastasis of LCNEC with a 3-mm tumor-free resection margin. The patient had a favorable clinical course without any complications, and he was discharged on postoperative day 6. There was no sign of recurrence 6 months after hepatectomy.

## 3 Discussion

Lung NETs account for approximately 25% of primary lung neoplasms [8]. In the 2015 WHO classification, lung NETs were classified into four categories: SCLC, LCNEC, carcinoid tumor and diffuse idiopathic pulmonary neuroendocrine cell hyperplasia. The diagnosis of LCNEC is based on recognition of both neuroendocrine morphology and the immunohistochemical demonstration of specific neuroendocrine markers, such as chromogranin, synaptophysin, and neural cell adhesion molecule, also known as CD56 [9].

Lung LCNEC is an aggressive and a rare type of lung cancer that accounts for 3% of all primary lung malignancies [1]. Even LCNEC patients with complete resection have poor prognoses with five-year survival rates of 15–57% and distant metastasis rates of 65% [3]. Therefore, surgery alone is not sufficient to treat patients with LCNEC, and subsequent adjuvant therapy may be necessary [10]. The clinical and biological characteristics of LCNEC are similar to those of SCLC [2], and Iyoda et al. reported that compared with the historical control patients, patients who selected cisplatin and VP-16 as adjuvant chemotherapeutic regimens after complete resection had a better prognosis in the prospective study [11]. Therefore, we also administered the same regimen as adjuvant chemotherapy, and there was no sign of recurrence 4 years after lung resection.

LCNEC with distant metastasis has a poor response to systemic chemotherapy [4], and the median survival of patients with distant metastasis is estimated to be approximately 6 months, with a five-year survival rate of 0% [5]. The common sites of metastasis from LCNEC are the liver, adrenal glands, bones and brain [6], but there are no previous reports of resection for metastasis of LCNEC. In our case, we performed hepatectomy for solitary liver metastasis from lung LCNEC, and there was no sign of recurrence 6 months after hepatectomy. This time was longer than the median survival time previously reported, suggesting a benefit of aggressive surgical resection for solitary distant metastasis of LCNEC.

Considering the high rate of recurrence after hepatectomy, we selected laparoscopic nonanatomical hepatectomy to maximize the remnant liver volume and prepare for future repeat hepatectomy. In laparoscopic hepatectomy, surgeons cannot use their hands to palpate the liver to search for additional tumors or their fingers to mark the resection margin. Recently, fusion ICG-fluorescence imaging, which enables real-time indication of pseudocolor-fluorescence signals on white color images, serve as a simple navigation tool that provides information on tumor location, segmental boundaries, and bile duct anatomy [12]. In particular, it may assist in the safe and accurate completion of laparoscopic nonanatomical hepatectomy for tumors located in the right posterior areas, which are considered relatively unfavorable due to the limited gross inspection and poor tactile feedback [13]. In our case, the tumor located at S7 was also removed with a safe margin using fusion ICG-fluorescence imaging.

## 4 Conclusion

In summary, we report a case of laparoscopic hepatectomy for liver metastasis of lung LCNEC. Recurrence-free survival for 6 months postoperative is a short duration but is longer than the median survival time previously reported. Although the prognosis of LCNEC with distant metastasis is extremely poor, these results suggest that surgical resection for solitary distant metastasis of LCNEC may improve prognosis.

## Funding

This study did not receive any specific grant from funding agencies in the public, commercial, or not-for-profit sectors.

## Ethical approval

Ethical approval was not obtained for this case report.

## Consent

Written informed consent was obtained from the patient for publication of this case report and accompanying images. A copy of the written consent is available for review by the Editor-in-Chief of this journal on request.

## Author contribution

Hisoka Yamane: Concept and design of study, drafting the manuscript, participation in the treatment and perioperative management of the patient, approval of final manuscript.

Sachiko Yoshida: Design of study, approval of final manuscript.

Toshihiko Yoshida: Design of study, approval of final manuscript.

Masayasu Nishi: Design of study, approval of final manuscript.

Takashi Yamagishi: Design of study, approval of final manuscript.

Hironobu Goto: Design of study, approval of final manuscript.

Dai Otsubo: Design of study, approval of final manuscript.

Akinobu Furutani: Design of study, approval of final manuscript.

Taku Matsumoto: Design of study, participation in the treatment of the patient, approval of final manuscript.

Yasuhiro Fujino: Design of study, approval of final manuscript.

Kazuyoshi Kajimoto: Review of pathological findings, approval of final manuscript.

Toshiko Sakuma: Review of pathological findings, drafting the manuscript, approval of final manuscript.

Masahiro Tominaga: Design of study, revision, participation in the treatment of the patient, approval of final manuscript.

## Registration of research studies

Not applicable.

## Guarantor

Hisoka Yamane accepts full responsibility for the work and the conduct of the case report, had access to the data, and controlled the decision to publish.

## Provenance and peer review

Not commissioned, externally peer-reviewed.

## Declaration of Competing Interest

All authors declare that they have no conflicts of interest in relation to this study.
